# Bullying in surgery: senior surgeons' views on systemic drivers across governance, culture and resources in Australia and Aotearoa New Zealand

**DOI:** 10.1108/JHOM-06-2025-0371

**Published:** 2025-10-16

**Authors:** Paul Gretton-Watson, Sandra G. Leggat, Jodi Oakman

**Affiliations:** School of Psychology and Public Health, College of Science Health and Engineering, La Trobe University, Bundoora, Australia; Faculty of Health Sciences, La Trobe University, Melbourne, Australia; Centre for Ergonomics and Human Factors, La Trobe University, Melbourne, Australia

**Keywords:** Workplace bullying, Systems theory, Resource dependence theory, Intersectionality, Surgical teams, Governance, Resources, Cultural norms, Psychological safety

## Abstract

**Purpose:**

This study explores how governance, cultural norms and resources contribute to workplace bullying in surgical teams across Australia and Aotearoa New Zealand. It draws on Systems Theory and the Resource Dependence Theory (RDT) to examine how these constructs interact to shape both the persistence of bullying and the conditions for reform.

**Design/methodology/approach:**

Qualitative data were collected through semi-structured interviews with 31 senior surgeons across diverse specialties and institutional contexts. Thematic analysis was conducted using NVivo, with findings mapped to Systems Theory and RDT.

**Findings:**

Bullying behaviours were linked to rigid hierarchies, fragmented governance and exclusionary norms. In public hospitals, resource volatility and team instability heightened strain and reduced accountability. In private settings, continuity was greater but oversight inconsistent. Weak feedback loops, disengaged leadership and informal power structures enabled harmful conduct. Intersectional factors, particularly gender, training background and career stage, disproportionately affected women and international medical graduates. Participants identified governance reform, inclusive leadership and structural accountability as critical levers for change.

**Practical implications:**

Reducing bullying requires attention to feedback systems, resourcing stability and organisational culture. Structural redesign, inclusive leadership and equity-focused governance may enhance psychological safety across surgical settings.

**Originality/value:**

By applying the Systems Theory and RDT, this study offers a structural account of bullying as a product of governance gaps, resource asymmetries and cultural norms rather than individual misconduct. It shows how these conditions interact to generate behavioural risk in high-stakes teams.

## 1. Introduction

Workplace bullying in healthcare is increasingly recognised as a symptom of systemic dysfunction rather than isolated misconduct (Acquadro Maran *et al.*, 2023; Hoel and Vartia, 2021; Crebbin *et al.*, 2015). In surgical settings, where high-stakes decision-making intersects with entrenched hierarchies, bullying is especially persistent and damaging (Gretton-Watson *et al.*, 2024; Keller *et al.*, 2020). Bullying in surgery has been linked to poor psychological safety, burnout and attrition, with implications for both clinician wellbeing and patient outcomes (Carlasare and Hickson, 2021; Paice and Smith, 2009; Rennie and Rudland, 2024; Branch *et al.*, 2020; Chadwick and Travaglia, 2017; Gretton-Watson *et al.*, 2024). Recent research shows that bullying often stems from organisational fragility – fragmented governance, poor communication and cultural resistance to change – rather individual misconduct (Anany *et al.*, 2023; Beale and Hoel, 2011; Cleary *et al.*, 2010).

Much of the literature focuses on individual behaviour, professional civility or interpersonal dynamics, often overlooking how organisational structures, cultural expectations and resource flows shape the environments in which bullying is more or less likely to occur (Gupta *et al.*, 2020; LaGuardia and Oelke, 2021; Osier, 2021). Formal interventions include the Royal Australasian College of Surgeons' Operating with Respect initiative (Coopes, 2016; Royal Australasian College of Surgeons, 2015; Gretton-Watson *et al.*, 2024b), yet evidence suggests these alone are insufficient without complementary structural and systemic reforms (Hutchinson and Hurley, 2013; Hutchinson *et al.*, 2010; Leape *et al.*, 2012).

Growing attention to organisational accountability, driven by shifts in accreditation standards, regulatory oversight and public scrutiny, has prompted investigation into systemic levers of workplace culture. High-profile social movements such as #MeToo and inquiries into health system misconduct have demanded visible and enforceable cultural reform (Cleary *et al.*, 2010; Anany *et al.*, 2023; Eberhart, 2022; Benmore *et al.*, 2018). This momentum calls for a more integrated understanding of how workplace conditions enable or constrain bullying behaviours, particularly in contexts like surgery, where power, autonomy and performance pressure converge.

This study responds by exploring how senior surgeons across Australia and Aotearoa New Zealand perceive the systemic, cultural and resourcing factors that shape workplace dynamics and behaviour.

### 1.1 Background

Defined by the Royal Australasian College of Surgeons as “persistent behaviour directed at an individual that is intended to cause humiliation, offence, intimidation, or distress” (Royal Australasian College of Surgeons, 2015 Link to the website.) workplace bullying is particularly entrenched in surgical environments (Huang *et al.*, 2018; Sarr, 2016; Crebbin *et al.*, 2015). Contributing factors include hierarchical governance, resource dependencies and entrenched cultural norms that resist reform. Even where overt bullying has declined, subtler forms, such as exclusion, marginalisation or strategic under-resourcing, remain (Branch *et al.*, 2020; Clements *et al.*, 2020; Halim and Riding, 2018; Pei and Alseidi, 2022).

Previous research has highlighted the cumulative toll of bullying on clinicians, team function, organisational performance and patient outcomes (Chadwick and Travaglia, 2017; Gretton-Watson *et al.*, 2024; Acquadro Maran *et al.*, 2023; Gupta *et al.*, 2020; Lever *et al.*, 2019). Within surgery, hierarchical deference, long-standing gender imbalances and the valorisation of stoicism have all been implicated in exclusionary behaviours (Halim and Riding, 2018; Liang *et al.*, 2020; Ling *et al.*, 2016). These dynamics are especially potent in surgical teams, where rapid decision-making and high-stakes performance often discourage dissent, feedback or accountability (Gupta *et al.*, 2020; Salin, 2021).

Addressing bullying in surgery requires moving beyond individual or interpersonal explanations toward a more systemic understanding of how surgical workplaces are structured and sustained. Despite growing investment in behavioural training and awareness initiatives, such as the Royal Australasian College of Surgeons' *Operating with Respect* program, persistent reports of incivility, exclusion and intimidation suggest that education alone is insufficient to drive lasting change (Ling *et al.*, 2016; Gillen *et al.*, 2017; Gretton-Watson *et al.*, 2024b). Bullying behaviours often reflect organisational norms, particularly where those norms are reinforced by ambiguous authority, entrenched hierarchies and tolerance for poor conduct (Forel *et al.*, 2018; Liang *et al.*, 2019; Branch *et al.*, 2020; Cleary *et al.*, 2010; Westbrook *et al.*, 2021). When governance fails to require workers to act decisively or instil consistent consequences, staff may perceive these responses as symbolic, further entrenching cynicism and mistrust. In contrast, clear accountability processes and more decisive responses to egregious behaviour have shown promise in recalibrating norms and improving psychological safety (Mannion *et al.*, 2015, 2017; O'Donovan and McAuliffe, 2020; Edmondson, 2018).

These constructs are analysed through two complementary theoretical lenses - Systems Theory and Resource Dependence Theory (RDT) – to interpret how institutional arrangements and resource dynamics influence behavioural norms.

### 1.2 Theoretical framing

This study applies theory as an interpretive tool linking empirical observations with organisational dynamics relevant to bullying in surgical workplaces. It adopts a multi-level analytical approach guided by Systems Theory and RDT to explore how organisational design, power and behavioural risk interact. Systems Theory focuses on structure, feedback and adaptability of surgical teams (Anderson, 2016; Anderson and McDaniel, 2000; Bertalanffy, 1968; Braithwaite, 2018; Braithwaite *et al.*, 2017b; Chandler *et al.*, 2016; Kwamie *et al.*, 2021). RDT highlights how control over valued resources, access to operating time, personnel or training, can reinforce power asymmetries and silence dissent (Ansmann *et al.*, 2021; Fernández-Castro *et al.*, 2017; Hillman *et al.*, 2009; Marchand and Durand, 2011; Pfeffer and Salancik, 1978). These frameworks inform the study's focus on three interrelated constructs: governance, cultural norms and resources. Together, they provide a scaffold for analysing how workplace behaviour is shaped, sustained or disrupted in high-stakes surgical systems.

Governance, cultural norms and resources are distinct but interdependent. Governance, refers not only to formal decision-making structures but also behavioural oversight clarity, leadership visibility and accountability processes (Braithwaite *et al.*, 2017b; Ansmann *et al.*, 2021; Mannion *et al.*, 2015).

Resources extend beyond material or staffing allocations to include access asymmetries, procedural control and relational stability, reflecting concerns raised in RDT about dependency and power (Ansmann *et al.*, 2021; Hodgins *et al.*, 2020; Hillman *et al.*, 2009; Hutchinson *et al.*, 2010). Cultural norms encompass implicit behavioural expectations and professional identity signals that influence inclusion, silence and challenge, particularly in relation to gender, hierarchy or training background (Hoel and Vartia, 2021; LaGuardia and Oelke, 2021; Liang *et al.*, 2019). Intersectionality, as introduced by (Crenshaw, 1991), highlights how overlapping social identities – such as gender, race and professional status – interact to shape vulnerability and access to power within organisational systems.

By anchoring the analysis in these three master constructs, this framing maintains clarity without reducing theoretical complexity. These interdependent domains are elaborated in Sections 2.1 and 2.2 through the lenses of Systems Theory and RDT.

## 2. Conceptual framework and research rationale

### 2.1 Systems theory: a structuring lens

Rooted in the work of Bertalanffy (1968), Systems Theory positions healthcare organisations as dynamic, interdependent systems. Disruption in one component – team composition, procedural norms or communication pathways – can trigger cascading effects across the system. Rather than viewing problematic behaviour as isolated or anomalous, Systems Theory directs attention to how repeated breakdowns in governance or role clarity create cultural risks. In surgical teams, where interdependencies are high and pressure is constant, this lens helps explain how bullying becomes normalised through patterned system failures (Braithwaite, 2018; Braithwaite *et al.*, 2017a; Westbrook *et al.*, 2018; Gretton-Watson *et al.*, 2025; Halim and Riding, 2018).

A central contribution of Systems Theory is its emphasis on feedback loops. These loops allow systems to self-correct, but when absent or compromised, dysfunctional behaviours persist (McNab *et al.*, 2020). Failures to escalate concerns, lack of structured debriefs and informal team turnover diminish opportunities for feedback, weakening collective accountability. Braithwaite *et al*. (2022) highlight the principle of emergence, showing how workplace cultures form through interactional dynamics not reducible to individual actions alone. Homeostasis, or the system's tendency to return to a status quo, may preserve established power structures even when harmful (Gretton-Watson *et al.*, 2025; Hodgins *et al.*, 2020). Durable change requires more than new policies – instead it demands attention to how systems adapt, resist or suppress reform.

Systems Theory also examines how intersecting factors – such as role seniority, gender or cultural background – interact with structural instability. This can amplify vulnerability, especially in systems with poor transparency or limited peer support (Crenshaw, 1991; Ojala and Nesdale, 2004; Webb *et al.*, 2016). It offers a multi-level view of workplace culture, bridging team operations and organisation-wide norms.

### 2.2 Resource dependence theory: power, scarcity and control

RDT (Pfeffer and Salancik, 1978; Salancik and Pfeffer, 1978) complements the Systems Theory in showing how control over valued resources shapes workplace dynamics. In surgical teams, access to resources, such as theatre time, support staff and training opportunities, is unequally distributed. Such imbalances affect power, dependency and cohesion. While the Systems Theory maps how such resources flow through complex organisational structures, RDT shows how asymmetrical control over these resources entrenches power relations and normalises tolerance of bullying (Pfeffer and Salancik, 1978; Hillman *et al.*, 2009).

Power in RDT is formal and enacted via the ability to control uncertainty (Hillman *et al.*, 2009). When junior clinicians, international medical graduates or nursing staff are dependent on others for access to key professional resources, they occupy structurally weaker positions – exacerbating marginalisation. Hillman (2009) found that bullying was linked to environments marked by unstable rosters, rotating teams and insufficient resourcing. Volatile resourcing adds stress, disrupts teamwork and blurs accountability (Ozturk, 2021; Fernández-Castro *et al.*, 2017; Marchand and Durand, 2011; Ansmann *et al.*, 2021).

Importantly, RDT also helps interpret differences between public and private sector contexts. While private hospitals may offer more predictable team arrangements and greater clinician autonomy, culture may not improve, particularly where oversight mechanisms are weak or incentives skew behaviour. In contrast, public hospitals often face acute resource constraints yet may have more robust regulatory frameworks (Ansmann *et al.*, 2021). RDT accommodates both conditions, allowing analysis of how scarcity, autonomy and dependency interact across institutional settings.

Together, the Systems Theory and RDT offer a dual-level framework for this study. The Systems Theory emphasises how interdependencies, feedback breakdowns and organisational design shape emergent behaviour. RDT sharpens focus on how material constraints and power asymmetries structure risk and resilience. Both support a more integrated understanding of bullying in surgery, not as individual pathology, but as a patterned outcome of system-level conditions and resource-based vulnerabilities.

Both the Systems Theory and RDT converge around the construct of governance, though through different conceptual mechanisms. From a Systems Theory perspective, governance represents the structural architecture through which roles, rules and feedback processes are coordinated to enable system stability and adaptation. Failures in governance, such as absent leadership, poor communication protocols or fragmented oversight, disrupt the system's capacity for self-regulation, allowing harmful behaviours to persist unchecked. In contrast, RDT conceptualises governance as a mechanism for managing power and dependency within organisational hierarchies. Here, governance structures function to regulate access to valued resources, enforce compliance and buffer against environmental uncertainty. Where these structures are weak or selectively enforced, power asymmetries are amplified, and accountability diminishes. Taken together, both theories position governance not merely as an administrative structure, but as a critical system-level function that either mitigates or enables bullying dynamics, depending on how it is designed, distributed and enacted.

### 2.3 Addressing gaps in the literature

In applying this dual-theory approach, the study addresses three interrelated gaps in the literature:

1. *Limited Insight into Senior Surgeon Perspectives.* Existing research prioritises the experiences of junior doctors, trainees and nursing staff – groups more visibly affected by bullying. In contrast, little is known about how senior surgeons interpret and influence workplace behaviour. Given their role in shaping team culture, clinical norms and operational practices, their perspectives are essential to systems-level analysis (Gretton-Watson *et al*., 2024b, 2025; Gianakos *et al.*, 2022).
2. *Insufficient Attention to Organisational and Systemic Factors.* Many studies treat bullying as a behavioural or relational issue, despite calls to examine the organisational, structural and resourcing contexts. This includes how team instability, governance practices, resource constraints and power imbalances interact in poor workplace conduct (Ansmann *et al.*, 2021; Braithwaite *et al.*, 2022; Cvenkel, 2020; Mannion *et al.*, 2023).
3. *Fragmented Use of Organisational Theory.* While organisational theory is used to explore elements of workplace bullying, few studies employ an integrated framework linking system structure with resource flows and power relations. Systems Theory and RDT offer complementary perspectives that, when combined, explain how bullying behaviours are patterned, persistent and embedded within wider organisational logics (Drabek and Merecz, 2013; Hillman *et al.*, 2009; Pfeffer and Salancik, 1978).

By addressing these gaps, this study frames bullying not as a failure of individual professionalism but as an outcome of how surgical teams are governed, resourced and shaped by cultural norms.

### 2.4 Research questions

Building on the systemic and resourcing complexities identified in previous studies, this paper draws on the perspectives of senior surgeons across Australia and Aotearoa New Zealand. It applies an integrated theoretical lens, led by Systems Theory and supported by RDT, to examine the structural, cultural and systemic enablers of bullying in surgical teams.

The study addresses two interrelated questions:

1. How do governance, cultural norms and resources interact to shape the emergence and persistence of bullying behaviours in surgical teams?
2. What organisational and systemic conditions are perceived to support respectful, inclusive and psychologically safe cultures in surgical environments?

These questions guide the study's design and analysis, emphasising actionable leverage points for change.

## 3. Methods

### 3.1 Study design

This study adopted a qualitative design to explore the systemic, cultural and resource-related drivers of workplace bullying in surgical teams across Australia and Aotearoa New Zealand. Qualitative methods are well-suited to examining sensitive and contextually embedded phenomena like bullying, where lived experience and perception are central to meaning-making (Creswell, 2023; Creswell and Poth, 2017; Patton, 2015). An interpretivist approach recognised that bullying behaviours and workplace norms as socially constructed through local cultures, professional hierarchies and institutional practices.

Semi-structured interviews elicited reflective accounts (Kvale, 2015). The interview guide was informed by prior healthcare bullying literature and the Systems Theory and RDT to examine workplace behaviours.

Reflexivity was maintained throughout, with the interviewer attentive to positionality, particularly in relation to the seniority of participants (Berger, 2015; Braun *et al.*, 2022; Byrne, 2022). Coding rigour and conceptual alignment were strengthened through collaborative analysis with other authors, enhancing analytical depth and mitigating interpretive bias (Tong *et al.*, 2007; Berger, 2015; Braun and Clarke, 2022).

### 3.2 Participants

Participants were senior surgeons who had completed the Royal Australasian College of Surgeons' (RACS) mandatory Operating with Respect (OWR) workshops between 2018 and 2019. Of 63 expressions of interest, 31 interviews were completed, lasting 22–65 min (mean 42.03 min). The sample included 19 men and 12 women from a range of specialties including general, orthopaedic, urological, paediatric, ENT, vascular and neurosurgery. Geographically, 26 participants were based in Australia and five in Aotearoa New Zealand.

Purposive sampling aimed to capture variation across gender, specialty and setting (public, private, rural, metropolitan). Most participants worked across both public hospitals and private practice, allowing insights into how clinical governance, team structure and institutional resourcing intersect across sectors.

While formal saturation was not pursued in a strictly positivist sense, interviews continued until recurring themes and patterns were evident, indicating adequate conceptual coverage (Guest *et al.*, 2006; Galvin, 2015; Hagaman and Wutich, 2017). Prior research suggests saturation is typically reached within 12–16 interviews for homogenous, single-site studies (Guest *et al.*, 2006; Galvin, 2015) and 20–40 for multi-sited or cross-cultural designs (Hagaman and Wutich, 2017). Our 31 interviews with senior surgeons across two national health systems fall within this range, supporting sample adequacy and rigour.

### 3.3 Procedure

Participants were recruited with the support of RACS through Surgical News advertisements and automated invitations to past OWR attendees. Consent was obtained through a pre-interview survey collecting demographic data and inviting participation in the interview phase. Interviews were conducted individually via secure video conferencing to accommodate geographic diversity and scheduling needs, a method recognised for its rigour and accessibility in qualitative healthcare research (Braun and Clarke, 2022; Bradley *et al.*, 2007).

Interviews followed a semi-structured protocol, eliciting reflections on governance structures (including clinical governance), team dynamics, institutional norms and instances of bullying or conflict. Participants were encouraged to reflect on recent experiences to limit recall bias and ground insights in contemporary practice (Tong *et al.*, 2007).

All interviews were audio-recorded with consent, transcribed verbatim and reviewed for accuracy. An audit trail and analytic memos supported transparency and reflexivity, offering a structured link between raw data and thematic development (Tong *et al.*, 2007; Berger, 2015). The primary researcher brought both academic and practitioner perspectives to the interviews. Reflexivity memos and peer debriefing were applied throughout to manage potential bias and power asymmetries.

### 3.4 Data analysis

Thematic analysis was conducted using a hybrid inductive-deductive approach (Braun and Clarke, 2022). Initial coding was inductive, allowing key concepts to emerge directly from the data. NVivo software was used to organise and compare codes across transcripts (Braun and Clarke, 2006; Lainson *et al.*, 2019).

In the second stage, emergent themes were mapped onto the guiding theoretical frameworks. Systems Theory guided the analysis of team dynamics, communication pathways and clinical governance (Braithwaite *et al.*, 2022). RDT provided a lens for examining how resourcing patterns – staffing, theatre access, administrative support – influence behaviour and power asymmetries (Ansmann *et al.*, 2021; Ozturk, 2021; Pfeffer and Salancik, 1978).

To ensure credibility and coherence, coded transcripts and theoretical interpretations were reviewed by two academic supervisors. This process enhanced rigour by challenging assumptions, refining themes and ensuring alignment with the study's interpretive aims (Tong *et al.*, 2007).

### 3.5 Ethical considerations

Ethical approval was obtained from La Trobe University (Approval Number HEC, 18308). Informed consent was obtained, and participants were advised of their right to withdraw at any stage.

To ensure confidentiality, identifiable data were removed during transcription and reporting. Participant codes replaced names. The study followed the Consolidated Criteria for Reporting Qualitative Research (COREQ) checklist to enhance transparency and rigour throughout data collection, analysis and reporting (Tong *et al.*, 2007).

## 4. Results

Senior surgeons described workplace bullying as closely tied to broader systemic, resource-related and governance conditions, rather than solely driven by individual behaviours or interpersonal conflict. Guided by Systems Theory and RDT, the analysis identifies interconnected structural and cultural patterns that reinforce or disrupt bullying dynamics. Illustrative quotes from senior surgeons are included throughout this section. Table 1 *Illustrative Quotes Mapped to Thematic Constructs: Governance, Cultural Norms and Resources* provides detailed thematic support and additional quotations across the three core constructs: governance, cultural norms and resources.

**Table 1:** Illustrative quotes mapped to thematic constructs: governance, cultural norms and resources

Subconstruct/Theme	Theoretical lens	Summary insight	Illustrative quote	Surgeon code
*Governance*				
Fragmented escalation pathways	ST	Lack of clear or effective escalation channels undermines accountability and enables misconduct to persist	“Escalation pathways exist on paper, but they're not trusted or followed. People go around them, or nothing happens.”	B71
Role ambiguity in leadership	ST	Unclear leadership responsibilities diminish behavioural oversight and reduce trust in governance systems	“We don't always know who's responsible for dealing with poor behaviour. It just floats around.”	A63
Lack of behavioural enforcement	ST	Failure to apply consistent behavioural standards creates conditions for bullying to go unchecked	“Certain individuals never face consequences. Everyone knows, but nothing happens.”	A68
Symbolic consequences and removal of offenders	RDT	Demonstrated consequences for misconduct shift cultural norms and signal institutional seriousness	“When that toxic person finally left, the whole atmosphere lifted. It changed overnight.”	B74
Informal influence in local environments	ST	Localised power structures evolve in governance vacuums, undermining formal accountability mechanisms	“In some places, it's just who's been there longest that calls the shots, not necessarily the best leader.”	B66
Inconsistent clinical governance integration	ST	Separation between clinical and administrative governance leads to blind spots in behavioural accountability	“Clinical governance doesn't always talk to HR or execs. It all feels very disjointed when there's a problem.”	C61
Governance gaps in regional and rural contexts	ST	Absence of senior medical oversight in rural areas allows problematic behaviours to go unchecked	“Out in the regions, there's no senior clinical oversight. People just do what they want.”	B77
Disengaged senior leadership	ST + RDT	Absence of active leadership reduces trust in processes and enables behavioural drift	“You never see the leadership until there's a disaster. That doesn't build much confidence in the system.”	C74
Tokenistic reporting systems	ST	Feedback systems are perceived as ineffective or performative, reducing engagement and voice	“You report something, and it goes into a black hole. People give up trying.”	A68
Executive indifference to surgical issues	ST	Executives are often seen as distant from surgical realities, weakening governance credibility	“The execs don't understand surgery “they're disengaged from what actually happens.”	B79
Unclear accountability chains	ST	Ambiguous role boundaries and unclear escalation pathways allow issues to fall through the cracks	“No one really owns the problem. Everyone assumes it's someone else's job to intervene.”	C71
Tolerance of repeated behavioural breaches	RDT	Perceived institutional tolerance of repeated misconduct erodes trust and emboldens poor behaviour	“There are people with years of complaints, but nothing changes. It's demoralising.”	B74
Behavioural issues minimised by governance structures	ST + RDT	Governance systems may downplay or rationalise misconduct due to fear of reputational risk or political cost	“They're more worried about bad PR than about fixing the culture.”	A75
Informal workarounds substituting for governance	ST	Where formal systems fail, staff rely on informal solutions, which can entrench power imbalances	“We've developed workarounds, but they depend on personalities. That's not a system.”	B66
*Cultural norms*				
Gatekeeping based on identity	ST + RDT	Those outside dominant identity groups often face exclusion from influence and opportunity	“It's harder if you're not white, male and trained here. Everything's harder.”	A65
Normalisation of intimidation	ST	Aggression is accepted as part of surgical identity, making it difficult to challenge	“Some surgeons just yell. It's seen as part of the job – no one questions it anymore.”	B74
Gendered expectations of leadership	RDT	Women leaders are scrutinised more harshly and must perform additional emotional labour	“If a woman calls something out, she's ‘difficult.’ If a man does it, he's ‘assertive.’”	C71
Historical professional archetypes	ST	Legacy images of the ‘ideal surgeon’ still shape norms about who belongs and how they should behave	“We're still unpacking decades of macho surgeon culture. It doesn't change overnight.”	A56
Marginalisation of IMGs	RDT	International graduates are often seen as ‘less than’ and excluded from key opportunities	“As an IMG, you're always trying to prove yourself twice as hard.”	C68
Tolerance of passive-aggressive behaviours	ST	Indirect hostility is rarely addressed and contributes to a toxic undertone	“No one swears or yells, but the sarcasm and coldness cut just as deep.”	B66
Professional prestige used to silence	RDT	Influential surgeons are often protected or excused, reinforcing double standards	“If they bring in money or prestige, their behaviour is overlooked.”	A63
Cultural backlash to reform	ST	Attempts to modernise culture can trigger resistance from those invested in the status quo	“You get pushback – ‘We've always done it this way.’ That's the hardest wall to shift.”	B71
Exclusion from informal networks	RDT	Much influence occurs outside formal structures, to which not all staff have access	“If you're not in the after-hours drinks crowd, you're not in the loop.”	C74
Stigma around vulnerability	ST	Expressing uncertainty or stress is viewed as weakness, discouraging help-seeking	“You're expected to be bulletproof. Admitting strain is still taboo.”	A59
Ritualised hierarchy and deference	ST	Respect is often tied to seniority, even when behaviour is poor	“The culture still tells you not to challenge someone more senior – no matter what they say or do.”	B68
Lack of visible consequences for misconduct	ST + RDT	Failure to address behavioural breaches reinforces norm of impunity	“People know who the bullies are. Nothing happens. That's the real problem.”	C71
Microaggressions as cultural baseline	ST	Casual dismissiveness or stereotype-based remarks are pervasive and go unaddressed	“It's not overt discrimination. It's death by a thousand cuts.”	A65
Emotional stoicism as a cultural norm	ST	Emotional detachment is valorised, discouraging relational leadership or empathy	“Showing too much care is seen as soft. That attitude still lingers.”	B79
*Resources*				
Uneven access to operating theatres	RDT	Control over theatre time creates power asymmetries that can be used to reward or punish	“If someone doesn't like you, you might find your list bumped again and again.”	A52
Administrative understaffing	ST	Lack of admin support leads to operational chaos, stress and interpersonal tension	“There's just not enough admin staff, so everything is rushed and mistakes happen.”	B81
Late and unpredictable list changes	ST	Unstable scheduling undermines efficiency and increases stress for all team members	“You can't run a safe theatre list if it changes last minute every week.”	C71
Public vs private system disparities	RDT	Private systems offer greater control and stability, reducing sources of conflict	“In private, I know my team and my theatre. In public, it's a lottery.”	B68
Lack of continuity in theatre teams	ST	Frequent rotation of team members inhibits trust and smooth collaboration	“You never know who you're going to get. It makes the whole process harder.”	A67
Access to support staff and equipment	RDT	Gatekeeping of resources is used to assert dominance and control others' effectiveness	“Some people get the best anaesthetist and tools. Others get leftovers.”	B66
Inequitable allocation of procedural opportunities	RDT	Control over high-value learning or work is a key mechanism of influence and exclusion	“Training cases are given out as favoursism, not based on merit or fairness.”	C74
Time pressure and overbooking	ST	Overloaded schedules create a high-stress climate that reduces tolerance and increases conflict	“There's no slack in the systemic if anything goes wrong, tempers flare.”	B79
Under-resourcing of regional hospitals	ST + RDT	Structural underinvestment leads to both resource scarcity and informal power concentration	“There are so few resources that it becomes all about who controls what little there is.”	A75
Inconsistent availability of allied health	ST	Lack of coordination across disciplines disrupts patient flow and increases intra-team frustration	“Physio or OT might not be available, so discharges get delayed, everyone gets frustrated.”	A59
Hidden costs of advocating for staff	RDT	Those who use their capital to support vulnerable staff may face retaliation or marginalisation	“I've spoken up for nurses before, but it's cost me politically.”	A63
Staff turnover driven by burnout	ST	High churn disrupts continuity, erodes team cohesion and amplifies system stress	“Every week someone new is leaving. No wonder the team feels fractured.”	C71
Scarcity as a justification for incivility	ST + RDT	Perceived lack of time or capacity is often used to excuse poor interpersonal behaviour	“People use stress as an excuse. But it's still unacceptable.”	B74
Lack of protected time for teaching and mentoring	ST	Mentoring is seen as optional, not valued or resourced, which limits knowledge sharing and weakens team resilience	“We're supposed to teach, but there's no time, no space, it just doesn't happen.”	B71

**Source(s):** Authors’ own work

### 4.1 Systems conditions that reinforce or disrupt bullying

This section addresses RQ1 by outlining the key governance and resource pressures contributing to bullying behaviours.

#### 4.1.1 Hierarchical dynamics and governance failures

Participants consistently acknowledged hierarchy as both functionally necessary and potentially problematic. In high-stakes environments, defined roles and leadership were essential for clarity and efficiency. However, when authority was exercised without accountability, it could suppress feedback, entrench fear and foster bullying. One senior surgeon observed, “Hierarchy is fine, essential even, but only if the people with power know how to use it responsibly. Without oversight, it becomes a shield for poor behaviour” (B60).

Weak clinical governance and insufficient leadership oversight allowed hierarchical imbalances to go unchecked. This was particularly evident in regional and rural hospital settings, where governance was often less developed or absent. As one participant noted, “In small rural hospitals, there's no HR presence, no performance reviews – it's just you and the culture that's been tolerated for decades” (C63).

Shifting team compositions amplified these vulnerabilities, making it difficult to challenge poor behaviour or maintain standards. As one participant explained, “You can't build a culture if the team is constantly dissolving and reforming. It creates silos, turf wars, and no shared accountability” (B74).

The absence of clinical governance leadership was linked to persistent inappropriate behaviour. One participant remarked, “There's a leadership gap that no one wants to fill. Everyone says, ‘it's not my problem,’ and so it becomes everyone's problem” (B81). Another added, “A medical leader should say, ‘This is our culture, and this is how we enforce it.’ That kind of clarity is missing” (B71).

Notably, surgeons highlighted the symbolic impact of removing persistently harmful colleagues. Where such interventions occurred, morale and workplace tone changed immediately and profoundly. As one surgeon described, “There are some surgeons who've had every chance. Re-education, mediation, mentoring – you name it. When they're finally removed, it's a seismic shift for the team” (A75). Another reflected, “It only took one senior person being removed for the whole place to feel different. People started speaking more freely – like the air was different” (B74).

#### 4.1.2 Communication breakdowns and feedback failures

Breakdowns in communication were understood as systemic and cultural vulnerabilities, not just interpersonal dynamics. Participants highlighted the absence of consistent feedback loops, inadequate role modelling and limited opportunities for reflective discussion. One surgeon noted, “We are still learning to talk to each other in useful ways. Most of us didn't grow up in a culture of collaborative reflection” (B68). Another reflected, “We do the morning huddles, sure – but often it's like ticking a box. It doesn't get to the actual issues in team behaviour or safety” (A77).

Inconsistent application of safety protocols across departments (e.g. situational awareness exercises used in ED but not in theatres) emerged as a missed opportunity to embed more collaborative and communicative team dynamics. As one participant observed, “We've done simulation and huddles in ED, but we've never done this in theatre. There's no reflection on how we actually operate as a team” (A67).

#### 4.1.3 Governance gaps and localised norms

Participants expressed concern that governance vacuums allowed localised workplace norms to take hold. This was especially pronounced in rural or regional hospitals, where oversight and accountability mechanisms were limited. As one surgeon explained, “In rural hospitals, they operate as their own little worlds, and behaviours develop without intervention from management” (A77). Another added, “Some places just don't have the structures to step in when something goes wrong, it's all based on personality and local dynamics” (C67).

Surgeons often described operating theatres as isolated environments where usual behavioural norms could be suspended, enabling problematic conduct to become entrenched. One noted, “Theatre becomes its own little world where behaviours are tolerated that wouldn't be allowed anywhere else” (B68), while another commented, “It's a closed loop. You wouldn't behave like that in front of a patient's family – but in theatre, you get away with it” (C79).

Participants repeatedly linked the absence of clear clinical governance leadership to ongoing behavioural issues, noting that when local leaders failed to set expectations, a drift into toxic norms became likely. One participant remarked, “Leadership sets the tone. If you don't have someone saying, ‘this is how we do things here’, things just unravel” (A64).

Conversely, where governance structures act decisively, symbolic interventions had powerful effects on team culture and morale. As one surgeon reflected, “When one senior person who was notoriously toxic left, the whole department just lifted overnight. It was like everyone could breathe again” (B74). Another stated, “Certain doctors who wreak havoc need to be weeded out … they're unresponsive even to re-education programs” (A75).

These accounts illustrate how governance failures not only enable bullying but also erode team trust and psychological safety. Local cultural drift, when left unchallenged, fosters environments where poor behaviours become normalised, especially in the absence of formal accountability mechanisms.

### 4.2 Resource dependencies and structural vulnerabilities

#### 4.2.1 Team continuity and operational efficiency

While team instability emerged as a vulnerability linked to governance breakdowns (see 4.1.1), participants also spoke about stable staffing as a critical resource enabler of operational efficiency, mutual respect and reduced stress – particularly in better-resourced private settings. Familiarity within surgical teams supported predictability, accountability and cohesion, while unstable rosters in public settings contributed to repeated disruptions and communication lapses. As one participant noted, “In the private system, I usually get the same anaesthetist, same operating theatre nurses. It makes an enormous difference to how smoothly things run” (B79). Another added, “The small private hospitals are more likely to have the same half-dozen nurses who've been there forever and know how I work. In public, I'd be lucky to see the same person twice” (A76).

One surgeon noted that these dynamics were not just operational but relational, pointing to stronger interpersonal rapport and mutual understanding as hallmarks of effective private teams: “I know everyone in the hospital. Our kids play together. You can't really piss anyone off – so people make more of an effort, and it shows in the teamwork” (D81).

These reflections reinforce how consistent team composition facilitates clinical flow and enables interpersonal trust. Participants described how familiarity within teams fostered informal communication, smoother decision-making and greater psychological safety. Constant staff turnover in public hospitals was perceived to erode collective efficiency, increase stress and make learning and conflict resolution more difficult.

#### 4.2.2 Autonomy, bottlenecks and status asymmetries

Surgeons in private practice reported high autonomy over scheduling, staffing and clinical decisions, enabling efficient workflows. In contrast, those in public hospitals experienced bureaucratic bottlenecks and limited influence over resource allocation. One surgeon noted, “In the private setting, I have fewer restrictions, more control over who I work with, and I can decide on what's best for my patient” (B68). Another explained, “Decisions are often made from above, with little input from those actually working on the ground, which disconnects the administrative goals from the operational realities” (A52). A third added, “In public, it's like flying blind: you don't know your team, you don't set the pace, and you spend more time reacting than operating” (C79).

These discrepancies often translated into unequal stress burdens, particularly when theatre access or staff availability was constrained. One participant observed, “There are simply not enough hands to support what we're expected to do – so we're stretched, stressed, and short-tempered” (A59). Another remarked, “When you're under pressure and still powerless to fix anything, that's when people start snapping” (B74).

#### 4.2.3 Protection and vulnerability in governance structures

Participants highlighted the need for governance systems that actively protect lower-status staff, such as junior doctors, nurses and administrative personnel, who felt they lacked the organisational support needed to challenge inappropriate behaviour across professional or hierarchical boundaries. While hierarchical dynamics shaped how power was expressed in day-to-day interactions (see 4.1.1), this theme focused on the structural safeguards – or their absence – that made speaking up risky or ineffective for those with less authority.

One surgeon noted, “You cannot expect a nurse to take on a powerful surgeon; the risks to their career are too great” (B71). The same participant added, “The low-power person can't moderate the behaviour of the high-power one. We need structural support for that” (B71). Another reflected, “You're asking the person with the least power to confront the person with the most – it's a system set up to fail” (A70).

Surgeons repeatedly emphasised the need for governance mechanisms, particularly within clinical governance, that ensured accountability regardless of professional seniority. One surgeon stated, “Governance needs to work whether the perpetrator is junior or a big name – it has to be fair and work both ways” (A68). Another noted, “We need policies that aren't just tick-boxes but are actually enforced, regardless of who the offender is” (B71). As one participant observed, “It has to be someone with power who calls it out. You can't rely on the most vulnerable person in the room to be the enforcer of respectful behaviour” (A56). A final reflection captured the broader concern: “Poor behaviour flourishes when leadership is weak. You need someone with the authority and courage to shut it down – and not just with a slap on the wrist” (C62).

#### 4.2.4 Intersectional access to resources and power

Although not the core focus of this study, several surgeons identified how gender, race and training background shaped access to institutional power, resources and leadership opportunities. These intersecting dynamics were described as persistent governance failures, contributing to cultural exclusion and differential vulnerability to bullying.

As one surgeon explained, “If you're a woman, or if you trained overseas, it's not just harder to get a job, it's harder to get heard” (A56). Another reflected, “Even if you're technically brilliant, if you're not in the inner circle, which is mostly white, mostly male, it's very hard to move up” (B68).

International medical graduates (IMGs) were often positioned as outsiders, navigating opaque rules and institutionalised gatekeeping. One participant noted: “IMGs are still not really part of the club. They get the harder jobs, less support, and almost have to prove twice over that they belong” (C63). In rural contexts, this was sometimes intensified by social isolation and lack of networked protection.

Women surgeons similarly reported exclusion from key decision-making processes and informal networks that shaped access to resources, theatre time and leadership pathways. Another added, “Some of the younger guys are really threatened when women push for change. They undermine you quietly, and it wears you down” (B74).

One surgeon reflected, “Governance helps, but only if it addresses how power works across gender and race, not just general professionalism” (A52).

Other participants pointed to how assumptions about commitment and leadership potential disadvantaged women and internationally trained surgeons. As one explained, “The culture still favours those who've had traditional, uninterrupted careers. If you've taken maternity leave or trained overseas, you're seen as ‘less committed’” (A67).

Another shared, “I've fought to bring more gender diversity to our team, but it's still a battle to convince others of its importance” (C71). Still, there were hopeful signs of change: “We're moving away from a traditional stereotype of who surgeons should be, and that brings a richer range of perspectives to the profession” (A56).

These comments reinforce the importance of considering equity in resourcing and oversight within surgical systems. Participants described a failure to embed equity considerations into governance frameworks, particularly around selection, support and career progression. When such inequities remain unaddressed, exclusionary norms can persist, limiting access to influence, safety and career progression for those outside dominant groups.

Together, these themes illustrate how bullying is sustained not only through interpersonal conflict but through systemic failures in governance, cultural inclusion and resourcing – issues explored further in the discussion that follows.

## 5. Discussion

**Figure 1:**
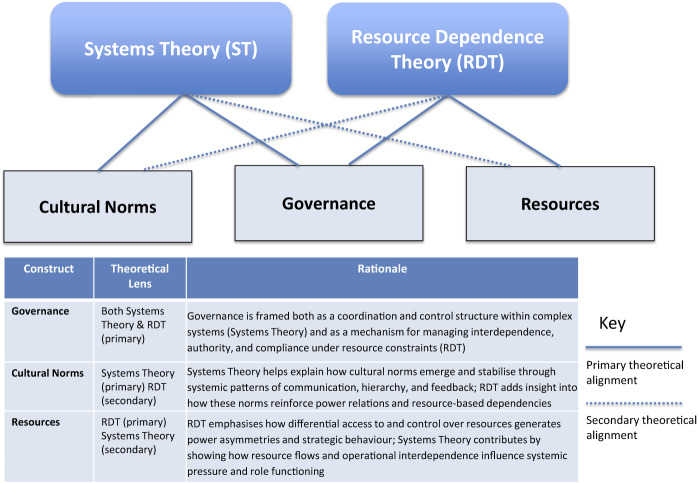
Conceptual framework linking systems theory and resource dependence theory (RDT) to structural drivers of bullying in surgery. Source: Adapted from Bertalanffy (1968) and Pfeffer and Salancik (1978)

This study found that senior surgeons in Australia and Aotearoa New Zealand perceive bullying within surgical teams to arise from deeper organisational systems rather than isolated interpersonal dynamics. Drawing on Systems Theory (Bertalanffy, 1968; Anderson, 2016) and RDT (Pfeffer and Salancik, 1978; Ansmann *et al.*, 2021), this discussion interprets the findings through the lens of the three core constructs identified in Figure 1: governance, cultural norms and resources. These constructs form the analytical framework for addressing the study's two research questions:

1. How do governance, cultural norms and resources interact to shape the emergence and persistence of bullying behaviours in surgical teams?
2. What organisational and systemic conditions are perceived to support respectful, inclusive and psychologically safe cultures in surgical environments?

The following sections respond to these questions in turn, integrating participant data with theoretical insight and relevant literature.

### 5.1 How do governance, cultural norms and resources interact to shape the emergence and persistence of bullying behaviours in surgical teams?

While a substantial body of research has examined incivility, microaggressions and interpersonal deviance (Cortina, 2008; Gretton-Watson *et al.*, 2024), this study adds value by exploring how those behaviours become tolerated or amplified through systemic conditions and governance failures.

Senior surgeons in this study described bullying not as the product of individual temperament or isolated misconduct, but as a behavioural adaptation to unstable, poorly governed and culturally exclusionary systems. Governance failures, resource asymmetries and exclusionary norms functioned together to enable and conceal disrespectful conduct (Dixit and Sambasivan, 2020; Hillman *et al.*, 2009; Fernández-Castro *et al.*, 2017; Ozturk, 2021).

This systems-based framing aligns with a growing literature that situates bullying as an emergent property of dysfunctional organisational structures rather than personal deviance (Hodgins *et al.*, 2020; Hutchinson *et al.*, 2010). While bullying behaviours may be enacted by individuals, they are often sustained and legitimised within systems marked by leadership drift, role ambiguity and structurally embedded power imbalances (Mishra *et al.*, 2021).

In larger hospitals, complex hierarchies, siloed responsibilities and inconsistent enforcement diluted accountability. In regional contexts, an absence of reliable clinical governance infrastructure – such as designated medical leadership or formal escalation pathways – left authority to be informally exercised through tenure, personality or local standing.

These accounts reflect what Systems Theory defines as degraded feedback loops, where critical system signals (such as behavioural breaches) do not generate timely or coordinated responses. Without effective feedback and correction mechanisms, bullying behaviours become embedded in daily routines and tacitly accepted. Braithwaite *et al*. (2022) emphasise how organisational microsystems that lack responsive feedback mechanisms drift toward dysfunction, reinforcing poor norms. Participants also described how siloed governance structures – dividing clinical and non-clinical oversight – created blind spots in behavioural risk management and enabled problematic subcultures to take root.

Resource constraints further compounded these dynamics. Surgeons working in public hospitals linked coercive behaviours and emotional volatility to chronic shortages of theatre time, staffing and administrative support. System stressors such as late list changes and understaffing heightened tensions, particularly where access to critical resources was unequally distributed. Those who controlled access to procedures or training held substantial influence, often exercised without procedural safeguards.

RDT describes this as dependency asymmetry: when one party controls access to a valued resource, those dependent on it are disempowered and less able to resist mistreatment. Fernández-Castro *et al*. (2017) argue that resourcing instability not only generates stress but undermines the ethical climate of healthcare organisations. In this study, fear of reputational damage or career limitation was commonly cited as a reason why junior staff did not report bullying. As one participant put it, “If your future depends on that person, you keep your mouth shut” (A63).

Cultural norms that reinforced dominance and conformity were also seen as central to the persistence of bullying. Participants described surgical environments in which assertiveness, competitiveness and stoicism were idealised, while behaviours that signalled vulnerability or divergence from dominant norms – especially among women and international medical graduates – were marginalised. Such norms were historically reinforced expressions of surgical identity (Liang *et al.*, 2019; Fox and Holland, 2024; Salin and Hoel, 2013; Gretton-Watson *et al.*, 2025).

This pattern is consistent with research on institutionalised professional cultures, where legitimacy and influence are maintained through implicit gatekeeping (Hoel and Vartia, 2021). Systems Theory refers to this as cultural coupling, where values, roles and behaviours become mutually reinforcing and resistant to change (McNab *et al.*, 2020). From an RDT perspective, exclusion from informal networks also means exclusion from influence, resources and advocacy, further entrenching asymmetric power structures (Ozturk, 2021; Ansmann *et al.*, 2021; Patterson *et al.*, 2018).

Together, these findings suggest that bullying becomes structurally embedded when governance systems are fragmented, resource access is politicised and cultural norms reward silence or conformity. In such environments, bullying emerges less as an aberration and more as a predictable adaptation to how power and risk are distributed within the system.

### 5.2 What organisational and systemic conditions are perceived to support respectful, inclusive and psychologically safe cultures in surgical environments?

While many participants described environments that enabled bullying, others identified structural features that fostered respect, inclusion and psychological safety – conditions known to mitigate bullying risk (Braithwaite *et al.*, 2017b; Cvenkel, 2020; Gretton-Watson *et al.*, 2025). These protective factors operated through governance clarity, resourcing stability and inclusive cultural norms.

Governance mechanisms were central to these safer environments. Participants described transparent escalation procedures, formal behavioural expectations and credentialing processes with conduct-based criteria. Clearly defined and consistently enforced systems gave participants confidence that poor behaviour would be addressed fairly. Leadership presence was also crucial: surgical and medical directors who were engaged, values-aligned and procedurally consistent were seen as essential to creating psychological safety and institutional trust.

These accounts align with Systems Theory, which characterises effective systems as those able to adapt to feedback and correct deviations from core norms (Edmondson, 2018; Sidani and Reese, 2020). Braithwaite *et al*. (2022) emphasise that relational engagement and distributed leadership are critical to sustainable cultural change, particularly in contexts where trust has previously been eroded.

Participants also emphasised the importance of stable resourcing. Predictable operating lists, consistent team assignments, reliable administrative support and protected time for training reduced friction and improved teamwork. Several participants noted that team continuity over time fostered mutual respect, informal accountability and a shared rhythm of work. RDT helps explain how stable access to valued resources reduces dependency-based power imbalances and promotes agency (Mishra *et al.*, 2021; Patterson *et al.*, 2018).

Cultural norms were equally important in enabling psychological safety. As Edmondson (2018) notes, psychological safety is not determined by personality but by environment, shaped by leadership cues and group dynamics. Participants in this study described how practices such as regular team briefings, cross-disciplinary input and leadership humility lowered status barriers and encouraged openness. In these environments, junior staff spoke up more readily and felt respected (Gillespie *et al.*, 2016).

These protective conditions were often mutually reinforcing. For example, inclusive leadership required procedural backing, and resource stability alone was insufficient without clear behavioural expectations. Such interdependence supports recent literature that argues cultural change is most durable when embedded in well-aligned systems and supported by feedback loops that reward and reinforce desired behaviours (Churruca *et al.*, 2022; Bernard and Geiger, 2024; Patterson *et al.*, 2018).

### 5.3 Implications for health system and hospital leaders

The findings of this study offer practical insights for health system leaders, hospital executives and surgical department heads seeking to reduce bullying and create sustainable cultures of respect.

First, behavioural expectations must be embedded into the foundations of clinical governance, including credentialing, accreditation and performance review systems. Participants stressed that policies without application were easily undermined. As Mannion *et al.* (2015, 2017) argue, governance must treat professionalism with the same seriousness as clinical quality to be credible and effective.

Second, resource allocation should reflect an understanding of how dependency structures shape workplace behaviour. Unstable rosters, inconsistent operating time and discretionary access to training were often linked with tension and coercion. Reducing these risks requires deliberate investment in team continuity, administrative support, and equitable access to procedural opportunities. RDT emphasises that where key resources are unequally controlled, vulnerability increases and voice diminishes (Ansmann *et al.*, 2021; Birken *et al.*, 2017). Ozturk (2021) further suggests that inclusive systems are not only culturally desirable but must also be materially resourced to endure.

Third, cultural change must be operationalised through mechanisms that promote accountability and flatten hierarchies. Participants identified structured peer review, routine team debriefs and reciprocal mentoring as useful tools for surfacing tensions and encouraging open dialogue. These mechanisms were valued not only for their functional benefits but for the symbolic signal they sent about shared responsibility and inclusion.

Finally, consequences for misconduct emerged as a pivotal concern. Several participants described longstanding frustration that individuals known to behave poorly were shielded from accountability due to seniority, revenue generation or institutional loyalty. This dynamic was fundamentally corrosive to trust and cultural credibility. Similar concerns are reflect in recent developments in the UK's National Health Service, where the removal of senior ambulance leaders following sustained bullying complaints was viewed as a critical turning point (Paduano, 2023). As Eberhart (2022) argues, symbolic actions play a vital role in re-establishing legitimacy within professional institutions. Structural interventions may create pathways for reform, but visible responses to misconduct determine whether values are genuinely upheld.

For surgical workplaces in Australia and Aotearoa New Zealand, governance systems must be both procedurally robust and publicly credible. Consequences should be applied fairly, consistently and transparently, regardless of a clinician's seniority or institutional standing. These findings also carry implications for medical education and continuing professional development. Embedding leadership and communication training within surgical curricula – particularly covering clinical governance, psychological safety and conflict resolution – may support the development of more respectful and inclusive team cultures.

### 5.4 Theoretical and practical synthesis

This study finds that bullying in surgical teams is shaped not only by interpersonal dynamics or individual traits, but by systemic conditions which reinforce or constrain poor behaviour. These conditions include the alignment and performance of governance systems, the stability and equity of resource access and the prevailing norms defining professional legitimacy.

Recent literature has established the influence of personality, group identity and team-level inclusion on bullying in surgery (Cope *et al.*, 2017; Gretton-Watson *et al.*, 2025; Bernard and Geiger, 2024; Bochatay *et al.*, 2019). Interactional styles, social identity and emotional regulation can increase vulnerability or resilience within high-stakes teams (Patterson *et al.*, 2018; Chandrasiri, 2017). This paper extends the explanatory arc by showing how these behavioural patterns are nested within broader institutional contexts.

Systems Theory highlights how bullying can become normalised when feedback mechanisms are weak, leadership is ambiguous or reporting systems are inconsistent (Reynolds *et al.*, 2020; Kwamie *et al.*, 2021; Chandler *et al.*, 2016; Bernard and Geiger, 2024). RDT explains how bullying can thrive when individuals hold unchecked control over valued resources while others remain silent due to fear of exclusion or career damage (Ansmann *et al.*, 2021).

Together, these frameworks explain not only when bullying occurs, but why it persists. Misaligned systems, politicised resources and exclusionary norms make silence and avoidance a rational responses to risk. Conversely, well-aligned systems, stable teams and visible accountability promote inclusion, voice, personal agency and respect.

As Edmondson (2018), and Braithwaite *et al*. (2017) argue, values alone are not enough to change culture. Structural integrity, procedural clarity and behavioural consistency are essential for psychological safety to take root. Without deliberate and visible system redesign, bullying may not only continue but become the rational outcome of an incoherent and permissive system. Recent literature on trauma-informed leadership (Bloom, 2013) supports the importance of relational safety and trust-building as organisational priorities. This resonates with participants' emphasis on procedural fairness, peer support and visible accountability.

These dynamics are not unique to Australasia. International studies have also linked operating room organisation, team stability and resourcing to surgical performance outcomes (Pasquer *et al.*, 2024) while demonstrated that governance capacity in healthcare organisations can be measured and strengthened using validated tools (Afşar Doğrusöz and Yazıcı, 2025). Such findings underscore the transferability of the present analysis and the value of comparative, cross-system learning.

### 5.5 Limitations

This study has several limitations. It draws exclusively on the perspectives of senior surgeons, providing valuable insight into leadership and system-level dynamics but excluding the experiences of junior clinicians or other health professionals affected by bullying. All interviews were conducted prior to the COVID-19 pandemic, so some governance, resourcing and accountability conditions may have since evolved. Contemporary examples referenced in the discussion – such as recent leadership interventions in public health systems – are intended as reflective context rather than participant-reported events.

As with all qualitative interview research, self-reporting carries risks of selective recall, attribution bias and social desirability. Participants may have downplayed their own involvement or framed experiences in ways favourable to professional identity. While this focus narrows triangulation across workforce levels, it also offers privileged insight into structural levers and governance processes not readily visible to junior staff.

Finally, while Systems Theory and RDT offered a productive framework for interpreting the data, other influences on bullying – such as psychological traits, interpersonal dynamics or broader institutional logics – also warrant consideration. Future studies might explore these dimensions to further explain how bullying endures or is challenged in surgical settings. Future research should explore how post-pandemic shifts in workforce governance and service pressures may have further shaped these dynamics.

## 6. Conclusion

This study contributes to the evolving understanding of workplace bullying in surgery by shifting analytical attention from individual and interpersonal conduct to the organisational and systemic conditions that shape it. This represents a shift from earlier research that has tended to emphasise behavioural remediation, offering instead a multi-level analysis that integrates structural, cultural and resourcing dimensions. Drawing on the perspectives of senior surgeons in Australia and Aotearoa New Zealand, the findings highlight how team instability, variable governance capacity, uneven resource access and leadership disengagement contribute to environments in which bullying becomes more likely and harder to address.

The application of Systems Theory and RDT reveals that bullying often reflects structural weaknesses rather than isolated interpersonal failings. Gaps in clinical governance, inconsistent feedback systems and workforce discontinuity can erode psychological safety and normalise disrespectful or exclusionary behaviour. These findings reinforce that workplace bullying in high-stakes environments such as surgery is often a foreseeable outcome of dysfunctional system design.

Importantly, the findings indicate that interventions centred solely on interpersonal training or generic frameworks are unlikely to yield lasting change. Instead, enduring change requires structural redesign, clear accountability mechanisms and leadership practices consistently reinforced across the organisation. Participants stressed the importance of governance systems that include transparent consequences for misconduct, even when this involves senior clinicians whose behaviour has previously gone unchecked. Visible enforcement was viewed as essential to rebuilding trust and signalling that professional conduct is non-negotiable. Accountability was framed not only as procedural, but also a symbolic act to restore cultural legitimacy.

Although focused on surgical teams, these dynamics are relevant to other high-stakes healthcare and emergency services settings as well as hierarchical, politically sensitive and resource-dependent environments. Understanding the interplay of structure and power is crucial for designing future interventions that treat bullying not as deviance but as a predictable outcome of governance failures, resource asymmetries and cultural drift. These findings support a shift from individualised blame toward systemic reform, offering a pathway for more accountable and respectful surgical workplaces. Integrated strategies that align governance, cultural norms and resources in coherent and enforceable ways are essential for lasting reform. Future research should test and refine these systemic levers across diverse clinical and high-stakes environments, building an evidence base for governance-driven cultural reform.
